# MYBA From Blueberry (*Vaccinium* Section *Cyanococcus*) Is a Subgroup 6 Type R2R3MYB Transcription Factor That Activates Anthocyanin Production

**DOI:** 10.3389/fpls.2018.01300

**Published:** 2018-09-11

**Authors:** Blue J. Plunkett, Richard V. Espley, Andrew P. Dare, Ben A. W. Warren, Ella R. P. Grierson, Sarah Cordiner, Janice L. Turner, Andrew C. Allan, Nick W. Albert, Kevin M. Davies, Kathy E. Schwinn

**Affiliations:** ^1^The New Zealand Institute for Plant and Food Research Limited, Auckland, New Zealand; ^2^The New Zealand Institute for Plant and Food Research Limited, Palmerston North, New Zealand; ^3^The New Zealand Institute for Plant and Food Research Limited, Motueka, New Zealand; ^4^School of Biological Sciences, The University of Auckland, Auckland, New Zealand

**Keywords:** blueberry, flavonoid, *Vaccinium*, anthocyanin, phenylpropanoid, MYB transcription factor, regulation, fruit

## Abstract

The *Vaccinium* genus in the family *Ericaceae* comprises many species, including the fruit-bearing blueberry, bilberry, cranberry, huckleberry, and lingonberry. Commercially, the most important are the blueberries (*Vaccinium* section *Cyanococcus*), such as *Vaccinium corymbosum* (northern highbush blueberry), *Vaccinium virgatum* (rabbiteye blueberry), and *Vaccinium angustifolium* (lowbush blueberry). The rising popularity of blueberries can partly be attributed to their “superfood” status, with an increasing body of evidence around human health benefits resulting from the fruit metabolites, particularly products of the phenylpropanoid pathway such as anthocyanins. Activation of anthocyanin production by R2R3-MYB transcription factors (TFs) has been characterized in many species, but despite recent studies on blueberry, cranberry, and bilberry, no MYB anthocyanin regulators have been reported for *Vaccinium*. Indeed, there has been conjecture that at least in bilberry, MYB TFs divergent to the usual type are involved. We report identification of *MYBA* from blueberry, and show through sequence analysis and functional studies that it is homologous to known anthocyanin-promoting R2R3-MYBs of subgroup 6 of the MYB superfamily. In transient assays, MYBA complemented an anthocyanin MYB mutant of *Antirrhinum majus* and, together with a heterologous bHLH anthocyanin regulator, activated anthocyanin production in *Nicotiana benthamiana*. Furthermore anthocyanin accumulation and anthocyanin structural gene expression (assayed by qPCR and RNA-seq analyses) correlated with *MYBA* expression, and *MYBA* was able to transactivate the *DFR* promoter from blueberry and other species. The RNA-seq data also revealed a range of other candidate genes involved in the regulation of anthocyanin production in blueberry fruit. The identification of *MYBA* will help to resolve the regulatory mechanism for anthocyanin pigmentation in the *Vaccinium* genus. The sequence information should also prove useful in developing tools for the accelerated breeding of new *Vaccinium* cultivars.

## Introduction

*Vaccinium* (family Ericaceae) includes many species with fruit eaten by humans, including blueberries, bilberry, cranberry, huckleberry, and lingonberry. The most important commercial species are the blueberries (*Vaccinium* section *Cyanococcus*), notable species of which are *Vaccinium corymbosum* (northern highbush blueberry) *Vaccinium virgatum* (rabbiteye blueberry), and *Vaccinium angustifolium* (lowbush blueberry). Driving market growth of this popularly called “superfood” is the increasing body of evidence around human health benefits resulting from the fruit metabolites, particularly products of the phenylpropanoid pathway such as anthocyanins (Davies and Espley, 2013; Norberto et al., 2013; Shi et al., 2017; Govers et al., 2018), which pigment the fruit. Blueberry species and cultivars show a great variety in the amounts and types of anthocyanins produced in the berries (McGhie et al., 2003; Lohachoompol et al., 2008; Stevenson and Scalzo, 2012; Li et al., 2017).

Central to the direct control of anthocyanin production is the MBW complex, consisting of R2R3MYB and bHLH transcription factors (TFs) and a WD-repeat (WDR) protein (Ramsay and Glover, 2005; Allan et al., 2008; Hichri et al., 2011; Davies et al., 2012). It is the R2R3MYBs that are generally the key factors in determining the spatial and temporal occurrence of anthocyanins (Borevitz et al., 2000; Schwinn et al., 2006; Albert et al., 2011). These are from subgroups (SGs) 5 and 6 of the plant R2R3MYB superfamily. Based on sequences currently available, SG6 types are characteristic of dicot species, but are also found in some non-grass lineages of monocots, while SG5 types are found in grasses and the orchid lineage of monocots (Paz-Ares et al., 1987; Ithal and Reddy, 2004; Hsu et al., 2015; Schwinn et al., 2016). In a rarity, both types have been reported to have roles in peach (*Prunus persica*), with SG6 types regulating pigment in flowers and fruits (Zhou et al., 2016) and an SG5 type reported to be functioning in petals of an ornamental variety (Uematsu et al., 2014). SG5 also contains R2R3MYBs that activate production of proanthocyanidins (PAs) and their polymers, condensed tannins. PAs are closely related to anthocyanins, and share common precursors in the flavonoid pathway. The SG5 PA regulator is exemplified by Arabidopsis TT2 (Nesi et al., 2001) and grape MYBPA2 (Terrier et al., 2009). There is also another phylogenetically distinct clade of R2R3MYB PA regulators, exemplified by grape PA1 (Bogs et al., 2007).

In recent years, genomics resources have been growing for the *Vaccinium* genus, including draft genomes for *V. corymbosum* (Gupta et al., 2015) and the cranberry species *V. macrocarpon*; Polashock et al., 2014), transcriptomics of developing fruit, and studies aimed at elucidating the control of phenylpropanoid/flavonoid production in the fruit (Li et al., 2012, 2016; Zifkin et al., 2012; Lin et al., 2018). Intriguingly, despite these studies, R2R3MYB SG6/SG5 activators of the anthocyanin pathway have not been identified in blueberries (Jaakola et al., 2010; Zifkin et al., 2012). Moreover, investigations into anthocyanin production in members of the *Myrtilis* section of *Vaccinium*, which unlike highbush blueberry and other members of the *Cyanococcus* section, have taxa with anthocyanin-colored berry flesh in addition to the skin, have resulted in conjecture that R2R3MYBs of the PA1 type are involved (Jaakola et al., 2010; Primetta et al., 2015; Zorenc et al., 2017). This includes *VmMYBPA1* (*VmMYB2*) from bilberry (*V. myrtillus*), which has an expression pattern that correlates with anthocyanin accumulation during berry development (Jaakola et al., 2010). *VmMYBPA1* transcript abundance is reduced in the green sections of fruit in which a berry developmental regulatory gene has been silenced (Jaakola et al., 2010) and in fruit of white-fleshed germplasm (Zorenc et al., 2017). Similarly, *MYBPA1* expression is reduced in white-fleshed berries of *V. uliginosum* (bog bilberry) (Primetta et al., 2015). However, a sequence from *V. corymbosum*, also named *MYBPA1*, was proposed to regulate PA and not anthocyanin production, based on the ability to activate promoters of two PA genes, but not that of an anthocyanin specific gene (Zifkin et al., 2012). Thus, there have been outstanding questions on the MYB TFs that control anthocyanin production in *Vaccinium*.

We present here the identification and characterization of an anthocyanin-promoting R2R3MYB SG6 sequence from blueberry that will help in resolving the regulatory mechanism for anthocyanin pigmentation in the *Vaccinium* genus. The sequence information should also prove useful in developing tools for the accelerated breeding of new *Vaccinium* cultivars.

## Materials and Methods

### Plant Material

Mature rabbiteye blueberry fruit skin and flesh were taken from pooled replicate samples harvested from Plant and Food Research in Motueka. Leaf anthocyanin and gene expression analysis was performed on newly emerged red or green leaves from glasshouse grown rabbiteye blueberry. Flowers from highbush blueberry were taken from the same glasshouse. For anthocyanin analysis, samples were freeze dried before extraction. For qPCR analysis, samples were flash frozen in liquid nitrogen before RNA isolation using the Spectrum^TM^ Plant Total RNA Kit (Sigma) according to the manufacturer’s instructions.

For RNA-seq, rabbiteye blueberry fruit from Plant and Food Research in Motueka, New Zealand, was flash frozen in liquid nitrogen. Three pooled replicate samples of the separated fruit skin and fruit flesh were processed for RNA isolation, as above. RNA-seq library preparation was conducted using the SENSE Total RNA-Seq Library Prep Kit (Lexogen) according to the supplied protocol. Input RNA was poly(A) selected using Dynabeads^®^ Oligo (dT)_25_ (Ambion^®^). RNA integrity was assessed using the Agilent RNA 6000 Nano Kit on an Agilent 2100 Bioanalyzer.

*Antirrhinum majus* plants were grown under standard glasshouse conditions in Palmerston North, New Zealand. The glasshouse was heated at 15°C and vented at 25°C, without supplementary lighting. The *rosea^dorsea^* line was used, which carries a non-functional allele of the R2R3MYB *Rosea1* (Schwinn et al., 2006).

### PCR Isolation of *MYBA*

RNA was extracted from whole pink immature fruit of highbush blueberry using the Spectrum^TM^ Plant Total RNA Kit (Sigma) according to the manufacturer’s instructions. The 3′-RACE PCR amplification (Frohman et al., 1988) used the primers K119 and “short primer” (SP) in the first round and the primers K115 and SP for nested PCR on the first-round products (see **Supplementary Table S1** for primer sequences). This generated several cDNAs putatively for the same gene that varied in the length of the 3′-UTR. The remaining 5′ section of the sequence was obtained with 5′-RACE PCR amplification using the Invitrogen 5′-RACE system. First strand cDNA synthesis used the primer K342, which is located in the 3′-UTR. First round PCR used K345 or K346 and the Abridged Anchor Primer (from the kit), followed by two subsequent rounds with the same specific primer and the Abridged Universal Amplification Primer.

### Phylogenetic Analysis

The deduced amino acid sequences were aligned using MUSCLE (Edgar, 2004) within Geneious (v 10.0.9) sequence analysis software (Kearse et al., 2012). For the maximum likelihood phylogeny, the aligned sequences were trimmed to include only the R2R3MYB region, because the C-terminal regions do not align between SGs of MYB proteins. The tree was constructed with PhyML (Guindon and Gascuel, 2003), with 1000 bootstrap replicates within Geneious. Sequences used and GenBank accessions: *Actinidia chinensis* AcMYB110 (AHY00342), AcMYB10 (PSS35990); *Arabidopsis thaliana* AtMYB4 (At4g38620), AtMYB12 (ABB03913), AtMYB75/PAP1 (AAG42001), AtMYB90/PAP2 (NP_176813.1), AtMYB113 (OAP11934.1), AtMYB114 (AEE34502.1), AtMYB5 (NP_187963.1), AtMYB123/TT2 (CAC40021); *Fragaria × ananassa* FaMYB1 (AAK84064.1), FaMYB10 (ABX79947.1); *Lotus japonicus* LjTT2a (AB300033); *Malus* × *domestica* MdMYB10 (ACQ45201); *Medicago truncatula* MtMYB5 (XP_003601609.3), MtLAP1 (ACN79541.1), MtMYB14 (XP_003594801.1); *Petunia hybrida* PhMYB27 (AHX24372), PhANTHOCYANIN2 (AAF66727.1), PhDEEP PURPLE (ADQ00393.1), PhPURPLE HAZE (ADQ00388.1), PhMYB4 (ADX33331.1); *Populus tremuloides* PtMYB134 (ACR83705.1); *Solanum lycopersicum* SlMYB12 (ACB46530.1); *Trifolium arvense* TaMYB14 (AFJ53053.1); *Trifolium repens* TrMYB4 AMB27079), TrMYB133 (AMB27081), TrMYB134 (AMB27082); *V. corymbosum* VcMYBA (this study: MH105054), VcMYBPA1 (JQ085966.1), VcMYB17 partial (ALP43798.1); *Vaccinium myrtillus* VmMYB2 partial (ADK79068.1); *Vaccinium uliginosum* VuMYBC2 (AKR80571), VuMYBPA1 (AKC94840.1); and *Vitis vinifera* VvMYBA1 (BAD18977), VvMYBA2 (BAD18978), VvMYBF1 (ACV81697), VvMYB5b (AAX51291), VvMYBC2-L1 (AFX64995.1), VvMYB-L2 (ACX50288.2), VvMYBPA1 (CAJ90831.1), VvMYBPA2 (ACK56131.1).

### Transient Transformation Assays

The vector *pSAK277* (Gleave, 1992) containing the *35S:VcMYBA* coding sequence with/without *35S:bHLH* was transformed into *Agrobacterium tumefaciens* strain GV3101 by electroporation followed by incubation on plate before infiltration. *Nicotiana benthamiana* plants were grown under glasshouse conditions using natural light with daylight extension to 16 h as previously described (Espley et al., 2007). Three leaves of 6-weeks-old *N. benthamiana* were used for infiltration and kept under the same growth conditions. Leaves were photographed and harvested 6 days after infiltration in liquid nitrogen and stored at -80°C until analysis.

*Antirrhinum* particle bombardment experiments using pKES8 were performed as described in Schwinn et al. (2016). pKES8 was formed by amplifying *VcMYBA* cDNA using primers K351 (AGT CGA ATT CAT GGA CAT AGT TCC ATT G), which added an *Eco*RI site next to the ATG that best fit the Kozak consensus sequence, and K352 (ACG TTC TAG AAG CGT AAC AAT CGA TGG A), which added an *Xba*I site to the end of 3′-UTR. The amplicon was digested and cloned into *Eco*RI/*Xba*I sites of pART7 (Gleave, 1992), putting it under the control of the *CaMV35S* promoter. The control was a *CaMV35S:GFP-ER* construct (Haselhoff et al., 1997), which localizes GFP to the endoplasmic reticulum. The white adaxial surface of the dorsal petals was bombarded using 300 or 400 kPa helium pressure. After bombardment, the petals were then cultured on half-strength MS medium (Murashige and Skoog, 1962) under 20–50 μmol m^-2^ s^-1^ light from Osram 36 W grolux fluorescent tubes (16 h photoperiod) at 25°C. At least two flowers were used for each construct per experiment, and each experiment was repeated at least twice.

### Promoter Activation Assays in Tobacco

Promoter fragments for *DFR* from Arabidopsis, apple, and rabbiteye blueberry, containing 1909, 1647, and 646 bp, respectively, upstream of the start code ATG of each candidate gene were isolated by PCR. Fragments were inserted into the cloning site of pGreenII 0800-LUC (Hellens et al., 2005) and modified to introduce an *NcoI* site at the 3′ end of the sequence. This allowed the promoters to be cloned as a transcriptional fusion with the firefly luciferase gene (*LUC*). The promoter-*LUC* fusion in pGreenII *0800*-*LUC* was used in transient transformation by mixing 100 μL of *Agrobacterium* strain GV3101 (MP90) transformed with the reporter cassette with 450 μL each of two other *Agrobacterium* cultures. These cultures had been transformed with cassettes containing a cDNA of *MYB* TF gene or a *bHLH* TF gene fused to the *35S* promoter, respectively, in either pSAK277 or pHex2 (Hellens et al., 2005). *N. benthamiana* growing conditions, *Agrobacterium* infiltration processes and luminescent measurements were as described by Hellens et al. (2005).

### Analysis of Transcript Abundance Using qPCR

Quantitative RT-PCR (qPCR) was used to measure transcript abundance for *MYBA* and *DFR* in five blueberry tissue types: berry flesh and skin (rabbiteye), green and red leaves (rabbiteye), and flowers (highbush). cDNA was synthesized according to the manufacturer’s recommendations (QuantiTect^®^ Reverse Transcription Kit, QIAGEN). qPCR was conducted using the LightCycler^®^ SYBR Green I Master (Roche LightCycler^®^ 480 System). Data shown are mean expression from four technical replicates of three biological replicates for each tissue type. Expression level reported is relative to the housekeeping gene *Actin*. Gene specific primers are listed in **Supplementary Table S1**. *Actin* and *DFR* primers are from Zifkin et al. (2012). Primer efficiencies were calculated using serial dilution. Reactions contained 2.5 μL Master Mix, 0.25 μL of each primer (10 μM), 1.25 μL diluted cDNA (1:25), and nuclease-free water (Roche Diagnostics) to a total volume of 5 μL, using reaction conditions previously reported (Espley et al., 2007) Analysis was performed using LightCycler software (Roche; version 1.5.0 SP4).

### HPLC Analysis of Anthocyanins

Approximately 500 mg of each sample of rabbiteye tissue (berry flesh and skin) was extracted in 5 mL ethanol: MilliQ: formic acid (80:20:1), with mixing at room temperature on a rotary shaker for 30 min, and incubation overnight at 1°C. Samples were brought to room temperature then centrifuged for 10 min at 3000 rpm; 500 μL from each sample were combined to create a composite sample for quality control. Samples were diluted either two or 10 times and 1-mL aliquots were analyzed by HPLC. The composite was diluted 10 times.

The HPLC system consisted of a Dionex Ultimate 3000 RS system: a SRD-3400 degasser, HPG-3400RS pump module, and WPS-3000 autosampler connected to a DAD-3000RS diode array detector under the control of Chromeleon software (version 7.2.3.7553, Thermo Fisher, United States). Separation was carried out on a Zorbax SB-C18 2.1 mm × 150 mm, 2.1-μm column (Agilent, United States) with the following gradient program: 0–0.5 min, 5% B; 0.5–10 min, 5–20% B; 10–15 min, 20–95% B; 15–16.5 min, 95% B; 16.5–16.8 min, 95–5% B; 16.8–20 min, 5% B. Solvent percentage was made up to 100% with A, where A = formic acid/MilliQ (5/95) and B = acetonitrile. The flow rate was 0.350 mL/min and the injection volume was 2 μL. Anthocyanin components were detected at 530 nm and peaks identified by comparison of peaks to authentic standards and previous reports of blueberry anthocyanins. The identifications were confirmed by mass spectrometry of a single sample.

Infiltrated leaf patches of *N. benthamiana* were harvested, weighed, and snap frozen in liquid nitrogen before freeze drying for 24 h. Leaf samples were then powdered, extracted, and analyzed by HPLC using the same protocol described for blueberry skin and flesh. Anthocyanin components were detected at 530 nm and quantified by comparison to authentic standards using a five point calibration curve.

### RNA-seq Differential Expression Analysis

Next-Generation Illumina sequencing with 100 bp paired end reads was performed by The Australian Genome Research Facility (Melbourne, Australia). Raw RNA-seq read data were pre-processed by trimming the raw reads (497,170,448 reads), filtering out contaminants, adapters, and low-quality base calls (<20), and quality checking (6.28% of reads filtered out as either homopolymer or <50 bp after clipping). The cleaned data were aligned to the gene models extracted from the published *V. corymbosum* genome^1^ using the .bed file for the genes provided. These alignments were counted (by fragment) and subjected to differential expression analysis using the DESeq2 analysis software package (Love et al., 2014). The resulting 48,637 differentially expressed genes were annotated with BLAST against *A. thaliana* proteins. To obtain results with known annotations of transcripts and/or genes, the cleaned data were aligned to the *V. corymbosum* transcriptome^2^, which is annotated, removing the requirement for annotation in the analysis. The counts from these alignments were again analyzed for differential expression with DESeq2, and the resulting 39,461 differentially expressed transcripts were combined with the annotations for the transcriptome. Notably, some transcripts in this transcriptome, and also in the set of the differentially expressed transcripts, did not have annotation provided. These will require manual annotation if required for further analysis. All data presented for differential gene expression were with a cut-off value of 2 log_2_fold.

## Results

### Identification of a Blueberry cDNA for an R2R3MYB Subgroup 6 Factor

BLAST analysis of an in-house *V. corymbosum* fruit EST library did not reveal a strong candidate for a R2R3MYB anthocyanin regulator. Therefore, 3′-RACE was used on cDNA from RNA derived from immature pink-colored whole fruit, using degenerate primers sited in the conserved MYB domain of characterized anthocyanin-related sequences. This amplified a candidate anthocyanin-related MYB sequence. The sequence was extended by 5′-RACE and a full ORF amplified. The gene was named *MYBA* (GenBank Accession MH105054). Transcript for *MYBA* was also present in subsequently obtained berryfruit RNA-seq data from rabbiteye blueberry (gene model 38459, **Table 2** and **Supplementary Figure S1**). Interestingly, our examination of the draft cranberry genome (Polashock et al., 2014) and transcriptome of its fruit (Sun et al., 2015) revealed *MYBA* to be present and expressed (**Supplementary Figure S1**), something that was not reported on in those publications.

**FIGURE 1:**
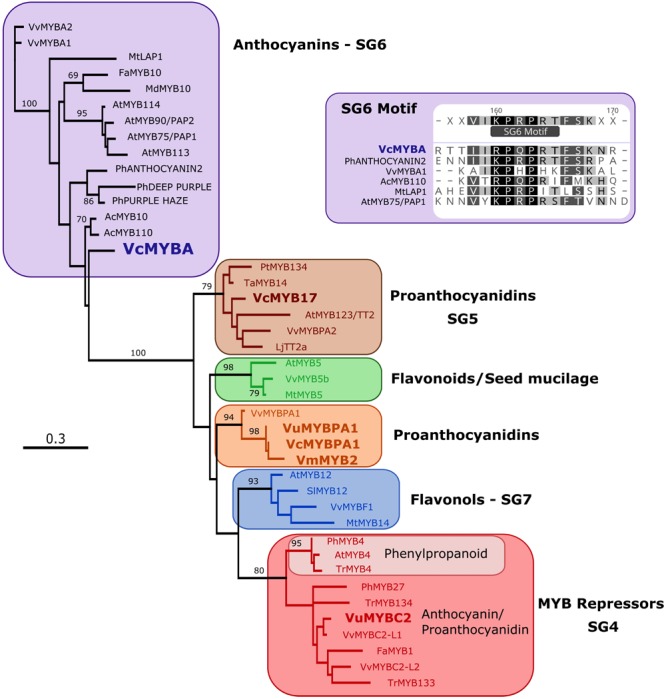
MYBA is a subgroup-6 R2R3MYB protein. A maximum likelihood phylogenetic tree of R2R3MYB sequences was generated using an alignment of the deduced amino acid sequences for the MYB domain. Sequences from *Vaccinium* species are in bold type. Node support > 65% from 1000 bootstrap replicates is shown. The inset shows that MYBA has the motif that is present in the variable C-terminal region of subgroup-6 anthocyanin regulators (Stracke et al., 2001; Schwinn et al., 2016).

Phylogenetic analysis was performed on *MYBA* and published *Vaccinium* R2R3MYB sequences against characterized flavonoid-related R2R3MYB sequences from other dicot species. The tree (**Figure 1**) was formed on an alignment of the deduced amino acid sequence for the MYB domain only. R2R3MYB sequences that activate the biosynthesis of different flavonoid classes separated into distinct clades, specifically for PAs (SG5), anthocyanins (SG6), flavonols (SG7), and R2R3MYB repressors (SG4). MYBA fell in SG6, while the previously identified *Vaccinium* sequences formed part of other clades: VuMYBC2 in SG4, VcMYB17 in SG5, and VmMYB2, VcMYBPA1, and VuMYBPA1 clustering with the grape sequence VvMYBPA1.

The MYB domain of *MYBA* contains the residues identified as being key for R2R3MYB interaction with bHLH proteins, including the motif [D/E]Lx_2_[R/K]x_3_Lx_6_Lx_3_R (Zimmermann et al., 2004; **Supplementary Figure S2**). Of the amino acid residues within the MYB domain that distinguish between R2R3MYB of SG5 and 6 (Lin-Wang et al., 2010; Schwinn et al., 2016), *VcMYBA* contained five of the six SG6 residues (**Supplementary Figure S2**), the exception being an N at position 82, which is also an N in *CmAN1*. Of the SG5/6 distinguishing amino acid residues within the variable C-terminal region (Stracke et al., 2001; Yamagishi et al., 2010; Schwinn et al., 2016), *MYBA* contained the SG6 residues (**Figure 1** and **Supplementary Figure S2**). In contrast, the published flavonoid-related R2R3MYB sequences from *Vaccinium, VcMYBPA1, VmMYBPA1*, and *VuMYBPA1*, have the SG5 characteristic residues in the MYB domain (**Supplementary Figure S2**).

A search of all non-redundant GenBank protein sequences gave a 97% identity score to a partial *V. corymbosum* sequence (ALP43799) that may be an allele of *MYBA* or a member of a gene family with *MYBA*.

### MYBA Activates Anthocyanin Synthesis *in planta*

**FIGURE 2:**
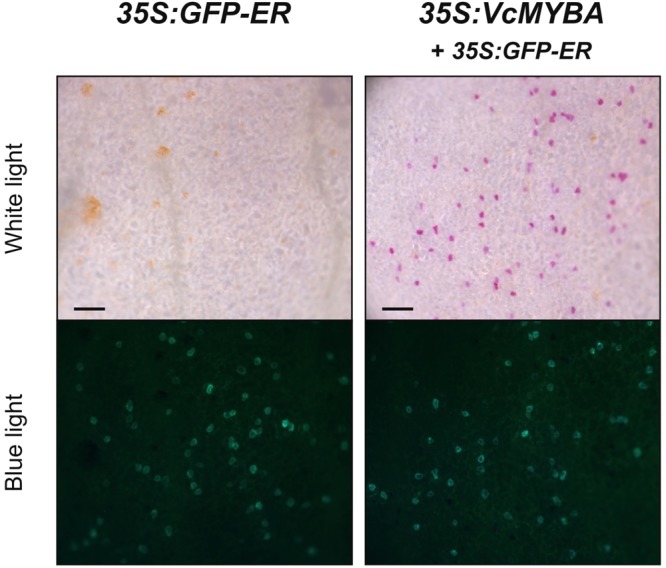
*MYBA* restores anthocyanin biosynthesis in *Antirrhinum rosea^dorsea^*. *VcMYBA* under the control of the *CaMV35S* promoter was biolistically introduced into white adaxial petal tissue of the *A. majus* line *rosea^dorsea^*, which carries a mutation in the main anthocyanin-promoting R2R3MYB *Rosea1*. Complementation is seen as restoration of pigmentation to single cells. *GFP-ER* was co-transformed as an internal control and viewed with blue light. Scale bar = 200 μm.

The phylogenetic analysis suggested that MYBA was associated with anthocyanin regulation. We tested the ability of *MYBA* to complement the *A. majus rosea^dorsea^* mutant (Schwinn et al., 2006), which has been used previously to test the activity of a range of SG6 anthocyanin MYB genes from diverse plant species (Albert et al., 2011, 2015; Schwinn et al., 2016). Biolistic transformation of *rosea^dorsea^* petals with *GFP-ER* alone was not capable of inducing pigmentation in transformed cells (**Figure 2**). Conversely, *MYBA* restored pink anthocyanin pigmentation to the transformed cells, which were identified by the GFP internal control (**Figure 2**). These assays were extended to a cultivar of highbush blueberry with white flowers. The transformation efficiency of petal cells was very low compared with that of *Antirrhinum*, as determined by the relatively infrequently occurring GFP-fluorescing foci. Nevertheless, while transformation with *GFP-ER* alone did not induce anthocyanin pigmentation, co-transforming with *35S:VcMYBA* resulted in some pink pigmentation (**Supplementary Figure S3**), suggesting that MYBA does regulate anthocyanin biosynthesis in highbush blueberry.

**FIGURE 3:**
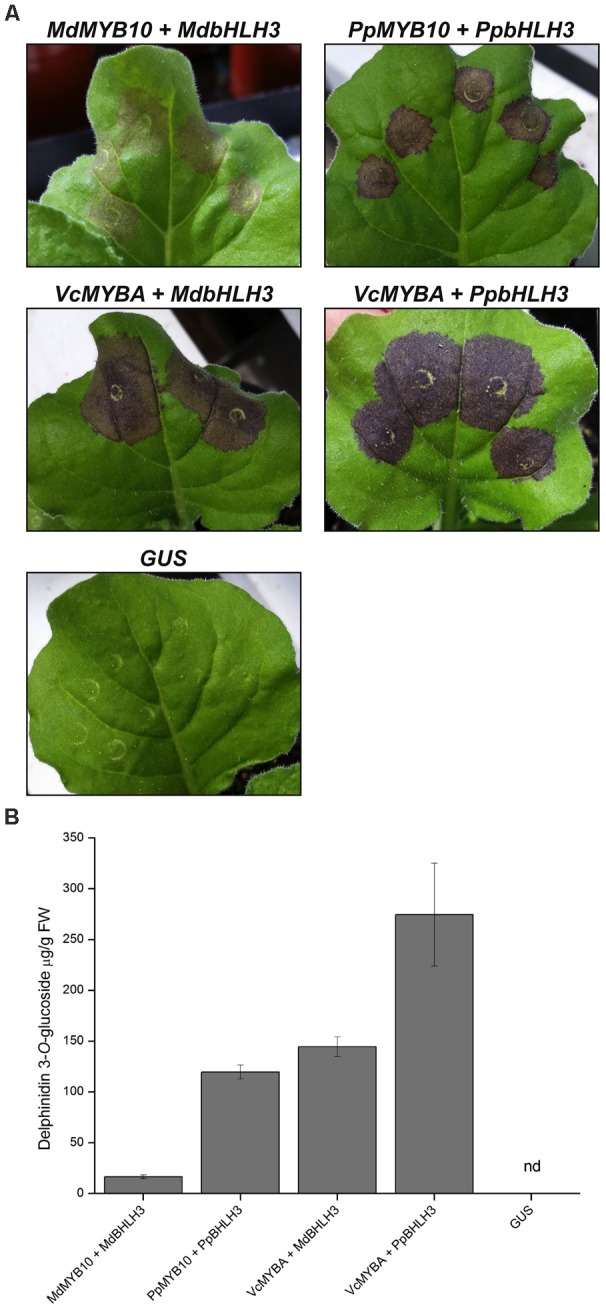
Heterologous expression of *MYBA* in *Nicotiana benthaminana* induces anthocyanin accumulation. **(A)** *Agrobacterium* infiltration of *N. benthamiana* leaves with SG6 R2R3MYB transcription factors *MdMYB10* (apple), *PpMYB10* (peach), or *VcMYBA* (blueberry) co-infiltrated with bHLH partners *MdbHLH3* or *PpbHLH3* induces pigmentation. *35S:GUS* was included as a negative control. **(B)** Anthocyanin quantification from infiltrated tissues. Nd = not detected. Mean of three biological replicates. Error bars represent ± SEM.

To further characterize the activity of MYBA, we performed transient *Agrobacterium* infiltration assays on *N. benthamiana* leaves (**Figure 3**). This landrace of *N. benthamiana* was used as it is capable of producing anthocyanin pigments when anthocyanin regulatory genes are expressed (Bally et al., 2015). In positive control experiments infiltration with the characterized apple anthocyanin regulator *MdMYB10* together with *MdbHLH3* resulted in purple pigmentation, and the intensity of the pigmentation was greater with the peach regulators, *PpMYB10* and *PpbHLH3*, than with the apple orthologs (**Figure 3A**). Infiltration of blueberry *MYBA* with either *MdbHLH3* or *PpbHLH3* resulted in even more intense purple pigmentation than in the positive control experiments. The intensity of coloration observed in the infiltrated leaves (**Figure 3A**) correlated with the amounts of anthocyanin pigments present in the tissue (**Figure 3B**). Over 95% of the anthocyanin in the leaves was identified as delphinidin 3-*O*-glucoside; however, a minor peak (λmax 536 nm) was also present on the chromatogram that may represent a second anthocyanin species, which could not be identified by comparison to the reference standards.

### *MYBA* Activates Promoters of a Key Anthocyanin Biosynthetic Gene

**FIGURE 4:**
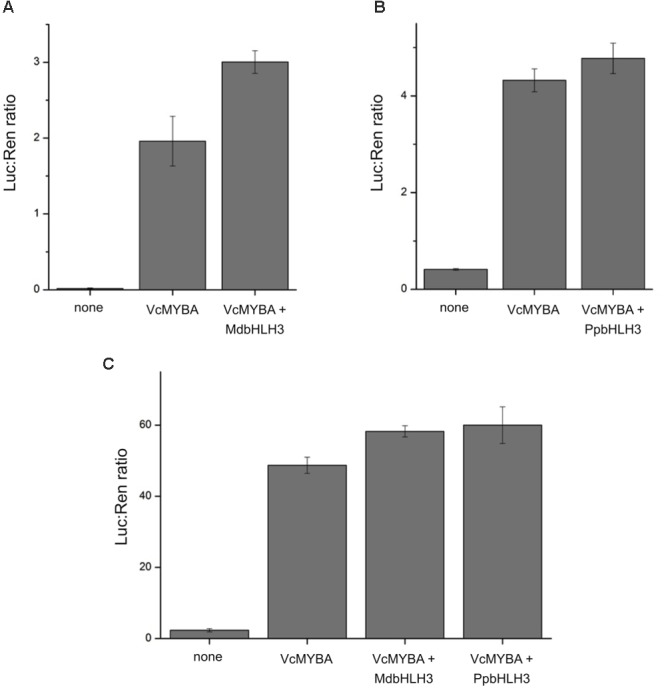
MYBA activates the promoter of *dihydroflavonol 4-reductase.* The *dihydroflavonol 4-reductase* (*DFR*) promoter sequences from **(A)** Arabidopsis (p*AtDFR*), **(B)** apple (p*MdDFR*), and **(C)** blueberry (p*VcDFR*) were isolated and cloned into dual luciferase reporter constructs. Promoter activation assays were performed by *Agrobacterium* infiltration of effector and reporter constructs into *Nicotiana benthamiana* leaves. Combinations of *VcMYBA* together with either *MdbHLH3* from apple or *PpbHLH3* from peach were tested. Mean of three biological replicates. Error bars represent ± SEM.

To test the functionality of *MYBA*, we used the dual luciferase promoter activation assay in *N. benthamiana*. We chose a key gene in the anthocyanin pathway, *dihydroflavonol 4-reductase* (*DFR*), and isolated promoters from Arabidopsis, apple, and blueberry, which were each fused to a luciferase reporter. Promoters alone were infiltrated into *N. benthamiana* to check for background activity and also co-infiltrated with *MYBA* alone or with known anthocyanin-associated bHLH factors from apple (*MdbHLH3*) or peach (*PpbHLH3*) (**Figure 4**). *MYBA* alone led to strong activation of all three *DFR* promoter fusions. The addition of *MdbHLH3* further increased this activation of the Arabidopsis promoter. However, the addition of bHLH did not appear to have an effect on the apple promoter (with *PpbHLH3*) or on the blueberry promoter, (with *PpbHLH3* or *MdbHLH3*). This suggests that *MYBA* is able to activate the *DFR* promoter from all three species without the dependence on a co-infiltrated bHLH cofactor. This is in contrast to apple MdMYB10, which is reliant on a co-infiltrated bHLH for *DFR* promoter activation (Espley et al., 2007). However, it is likely that endogenous *N. benthamiana* bHLH TFs (Montefiori et al., 2015) act as the co-factor with MYBA to drive *DFR* activation. These results demonstrate that MYBA is able to strongly activate the *DFR* promoter from blueberry and is capable of recognizing the promoters from heterologous hosts.

### Anthocyanin and Phenylpropanoid Biosynthetic Genes Are Differentially Expressed in the Skin and Flesh of Blueberry Fruit

**FIGURE 5:**
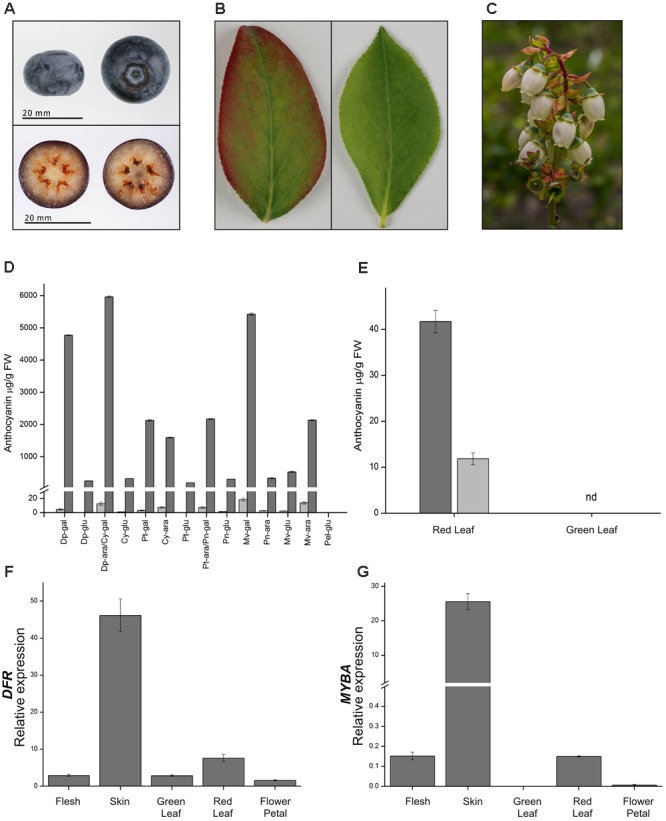
Anthocyanin accumulation and gene expression in blueberry fruits, leaves, and flowers. **(A)** Representative berry as used at mature stage. **(B)** Representative leaves at the same developmental stage showing presence (left) or absence (right) of anthocyanin pigmentation. **(C)** Flowers showing little, if any, anthocyanin. **(D)** Anthocyanin composition and concentration for fruit flesh (light gray bars) and skin (dark gray bars). Cy, cyanidin; Pn, peonidin; Dp, delphinidin; Pt, petunidin; Mv, malvidin; ara, arabinoside; gal, galactoside; glu, glucoside; nd, not detectable. Compounds Dp-ara/Cy-gal and Pt-ara/Pn-gal were not distinguishable at the resolution used for this analysis. **(E)** Anthocyanin composition in red and green leaves, cyanidin 3-galactoside (dark gray bars), and cyanidin 3-*O*-glucoside (light gray bars). Relative gene expression of **(F)** *DFR* or **(G)** *MYBA* in fruit flesh and skin, green and red leaves, and flower petals. Mean of three biological replicates. Error bars represent ± SEM. Rabbiteye and highbush blueberry used for **A/B** and **C**, respectively.

The fruit of blueberries (highbush and rabbiteye) are rich in anthocyanins, but this is restricted to the fruit skin, with the flesh tissues containing only trace amounts (**Figure 5A**). To gain additional information on anthocyanin production in the fruit and to identify other genes possibly involved in anthocyanin regulation, RNA-seq analysis was conducted on skin and flesh samples of rabbiteye blueberries, and differentially expressed genes were identified using DESeq2. Phenylpropanoid structural genes that provide precursor for anthocyanin production along with all the core structural genes in the anthocyanin biosynthesis pathway showed greater than 2 log_2_fold differences (**Table 1**). This was apparent from the first precursor step of *phenylalanine ammonia-lyase* (*PAL*) onward, with a number of gene models showing between 4 and 5.8 log_2_fold increase in fruit skin compared with flesh. These increases included *cinnamate 4-hydroxylase* (*C4H*) and *4-coumarateCoA ligase* (*4CL*), representing the next steps after PAL and which together provide the precursor products for use by the first committed enzyme in the flavonoid pathway, *chalcone synthase* (*CHS*). *CHS* produces naringenin chalcone and showed as much as 5.7 log_2_fold difference. The subsequent isomerization of naringenin chalcone by *chalcone isomerase* (*CHI*) to produce naringenin showed a 3.4 log_2_fold difference. The hydroxylation steps performed by *flavanone 3-hydroxylase* (*F3H*), which converts naringenin to dihydroflavonol, and the B-ring hydroxylases *F3′H* and *F3′5′H*, which determine the type of dihydroflavonol formed, were also well represented in the upregulated gene set. Two gene models for *DFR*, which reduces dihydroflavonol to leucoanthocyanidin, were also shown to be strongly upregulated in the skin, as was *leucoanthocyanidin dioxygenase* (*LDOX*), which produces anthocyanidin. One gene model for the glycosylation step to anthocyanin, *uridine diphosphate (UDP)-glucose:flavonoid 3-O-glucosyltransferase* (*UFGT*), was upregulated by 2.3 log_2_fold in fruit skin.

**Table 1:** Comparative abundance of transcripts encoding flavonoid and phenylpropanoid biosynthetic genes between skin and flesh of blueberry fruit.

Gene model	Average flesh	Average skin	Log_2_fold	*p*-value	Best Arabidpsis blast match (or other if poor match)
36273	0.3	294.0	7.72	1.99E-30	*Camellia sinensis*, flavonoid 3′,5′-hydroxylase (F3′,5′H). (AT5G06900)
38016	286.3	18289.0	6.15	0	AT4G22880.1, ANTHOCYANIDIN SYNTHASE (ANS, LDOX)
39197	1247.0	71130.7	5.99	0	AT4G22880.1, LDOX, TT18
7214	14.7	944.3	5.98	7.15E-75	*Populus trichocarpa*, F3′,5′H (AT5G07990.1, TT7)
39241	67.0	3587.0	5.87	0	AT5G13930.1, chalcone synthase (CHS, TT4)
24097	81.0	4548.7	5.80	7.97E-66	AT2G37040.1, phenylalanine ammonia-lyase (AtPAL1)
25738	370.7	17356.7	5.70	0	AT5G13930.1, CHS
32663	68.3	3082.0	5.62	3.01E-271	AT4G22880.1, LDOX
36277	0.3	66.3	5.38	2.26E-11	*Populus trichocarpa*, F3′,5′H (AT5G07990.1)
3392	74.3	3274.0	5.37	2.46E-37	AT2G37040.1, PAL
13816	247.7	6724.0	4.91	0	AT2G37040.1, AtPAL1
8284	282.0	7233.0	4.83	0	AT3G53260.1, AtPAL2
32449	1.7	82.0	4.73	6.59E-11	AT2G30490.1, cinnamate 4-hydroxylase (C4H)
7478	1541.7	28419.7	4.36	0	*Vitis vinifera* F3′H (AT5G07990.1, TT7)
7549	3637.3	61963.3	4.25	0	AT5G13930.1, CHS (TT4)
32451	693.7	11460.3	4.20	0	AT2G30490.1, C4H
36275	0.7	34.7	3.98	0.00000429	*Vitis vinifera* F3′,5′H-2-like, (AT5G07990)
24099	6.7	123.7	3.96	1.76E-12	AT3G10340.1, AtPAL4
33001	51.0	649.3	3.77	2.78E-46	AT3G51240.1, flavanone 3-hydroxylase (F3H)
6667	2434.7	23754.0	3.45	0	AT3G51240.1, F3H
34918	5.3	79.0	3.40	0.00000157	AT3G63170.1, chalcone isomerase (CHI)
1248	1788.0	15398.7	3.27	0	AT5G42800.1, dihydroflavonol 4-reductase (DFR, TT3)
17397	295.7	2543.7	3.24	1.2E-92	AT5G13930.1, CHS (TT4)
32630	1030.7	8589.3	3.21	5.37E-217	AT5G42800.1, DFR (TT3, M318)
842	1363.7	10223.3	3.06	3.86E-192	AT5G13930.1, CHS (TT4)
30312	2316.3	16659.7	3.00	8.55E-284	AT3G21240.1, coumarate:CoA ligase 2 (4CL2, AT4CL2)
18037	894.3	6141.0	2.93	2.83E-96	AT5G13930.1, CHS (TT4)
32793	22.7	158.0	2.67	0.00000479	AT5G08640.1, flavonol synthase 1 (FLS, ATFLS1)
592	2.0	26.0	2.62	0.003348852	AT1G61720.1, NAD(P)-binding Rossmann-fold superfamily (BAN)
15090	370.7	1710.3	2.35	1.95E-46	AT5G17050.1, UDP-glucose: flavonoid 3-*O*-glucosyltransferase
33905	331.0	47.0	–2.58	9.7E-16	AT5G54160.1, flavonol 3-*O*-methyltransferase

*RNA samples were compared using RNA-seq. Thirty candidate flavonoid biosynthetic genes had higher transcript abundance in the skin sample (>2 log_*2*_fold change), while one had higher transcript abundance in the flesh sample (>-2 log_*2*_fold change). Gene model number is abbreviated with full number shown in **Supplementary Table S2**. Data shown are genes with differential expression with a cut off value of greater than 2 log_*2*_fold.*

### RNA-seq Identifies Additional Candidates for Anthocyanin Regulation

Results are presented in **Table 2**. The most differentially expressed MYB TF in the dataset was MYBA (gene model 38459; **Supplementary Figure S1**), which was present at 97 fold higher in skin (a 6.6 log_2_fold differential). This further supports a role for *MYBA* in anthocyanin production. A gene ontology search for other TFs differentially expressed in the fruit skin and flesh revealed a number of transcripts corresponding to different TF families. Of these, 38 were more highly expressed in skin than in flesh and 17 higher in flesh. Of interest was the highly differentiated gene model 17527, which showed closest BLAST match to *At1G22640*, which encodes AtMYB3. In Arabidopsis, this SG4 repressor motif-containing R2R3MYB has been shown to be an active repressor of phenylpropanoid biosynthesis, acting via direct repression of the *C4H* gene, and its overexpression reduces anthocyanin accumulation (Zhou et al., 2017). Other highly differential MYBs included genes probably homologous to *AtMYB94*, involved in wax biosynthesis regulation; *AtMYB5*, a repressor of trichome branching; *AtMYB36* and *AtMYB58*, both associated with lignin regulation; and *AtMYB60*, involved in the light-induced opening of stomata.

**Table 2:** Comparative abundance of transcripts corresponding to transcription factors between skin and flesh of blueberry fruit.

Gene model	Average flesh	Average skin	Log_2_fold	*p*-value	Best Arabidpsis blast match (or other if poor match)
38459	1.7	252.3	6.6	1.68E-30	AT1G56650.1, PAP1, AtMYB75
22397	0.7	116.3	6.0	1.29E-17	AT4G21750.1, ATML1, MERISTEM LAYER 1
20712	103.3	4959.0	5.7	4.4E-209	AT3G47600.1, MYB94 – WAX production
17527	54.7	2449.7	5.6	2.71E-209	AT1G22640.1, AtMYB3
38996	51.0	2379.3	5.3	2.67E-29	AT3G13540.1, AtMYB5 – repressor
19022	26.7	1017.3	5.3	6.56E-97	AT3G13540.1, AtMYB5 – repressor
5243	55.3	2033.3	5.3	2.46E-182	AT5G46880.1, HOMEODOMAIN GLABROUS 5
3869	142.3	3059.3	4.4	4.18E-26	AT4G32890.1, GATA transcription factor
30629	2.3	76.3	4.3	6.09E-10	AT4G00050.1, bHLH phytochrome interacting factor7 (PIF7)
35094	0.0	32.0	4.3	0.00000315	AT5G15150.1, homeobox-leucine zipper protein ATHB-13-like
19290	62.7	1063.0	4.2	2.56E-84	AT1G16060.1, AP2/ERF domain ABA signaling
24778	11.3	208.7	4.1	4.12E-23	AT5G57620.1, AtMYB36
11854	1.3	42.3	3.9	0.000000943	AT2G46130.1, AtWRKY43
36826	34.3	490.3	3.9	1.99E-34	AT4G00730.1, ANTHOCYANINLESS 2 homeobox-leucine zipper
16653	1.7	43.3	3.8	0.000000859	AT4G21750.1, homeobox-leucine zipper ATML1
24402	16.3	204.7	3.6	1.87E-18	AT5G23730.1, WD40 repeat-like superfamily protein
19400	219.0	2363.0	3.6	3.92E-121	AT1G62300.1, WRKY transcription factor 6
24659	3.0	277.0	3.4	0.000397217	AT1G16490.1, AtMYB58
36111	971.3	8627.7	3.3	5.01E-186	AT1G62300.1, WRKY transcription factor 6
16197	7.7	101.3	3.3	0.00000436	AT1G67260.1, TCP family transcription factor (TCP1)
17058	7.7	93.3	3.2	0.00000137	AT4G04890.1, protodermal factor 2 (PDF2)
18053	47.0	402.3	3.2	5.63E-23	AT1G69690.1, transcription factor TCP15
12337	0.0	14.7	3.2	0.001119994	AT5G13180.1, NAC domain containing protein 83 (ANAC083, VNI2, NAC083)
13867	11.0	212.3	3.1	0.000314101	AT3G23240.1, ethylene response factor1 (ERF1, ATERF1)
11658	9.3	94.3	3.1	0.00000379	AT4G21440.1, MYB-like 102 (ATMYB102, ATM4, MYB102)
23031	4.3	51.7	2.9	0.00039243	AT4G17500.1, ethylene responsive element binding factor 1 (ATERF-1, ERF-1)
13951	3.3	45.0	2.9	0.000554601	AT5G11590.1, integrase-type DNA-binding superfamily protein (TINY2)
34102	0.0	17.0	2.8	0.004391771	AT5G50570.1, squamosa promoter-binding protein-like (SPL13A, SPL13)
19965	140.3	841.0	2.7	2.57E-29	AT1G08810.1, AtMYB60
24137	0.0	12.7	2.6	0.007613296	AT3G13840.1, GRAS family transcription factor
5570	9.3	66.0	2.6	0.0000657	AT4G27950.1, cytokinin response factor4 (CRF4)
3764	320.3	1645.3	2.5	3.1E-63	AT4G00050.1, bHLH PIF7
16000	376.0	1895.0	2.5	3.6E-74	AT4G00050.1, bHLH PIF8
36471	237.0	1168.3	2.4	7.31E-45	AT1G09250.1, bHLH DNA-binding superfamily protein
18515	1.3	22.7	2.4	0.011126278	AT5G22380.1, NAC domain containing protein 90 (anac090, NAC090)
25443	172.0	785.3	2.3	1.19E-23	AT1G62300.1, WRKY family transcription factor (WRKY6, ATWRKY6)
19599	51.0	229.3	2.2	4.72E-10	AT3G07340.1, bHLH DNA-binding superfamily protein
28899	24.7	115.7	2.2	0.0000114	AT2G20180.1, phytochrome interacting factor 3-like 5 (PIL5)
34686	23.3	2.3	–2.1	0.023373296	AT1G61660.1, bHLH DNA-binding superfamily protein
27916	10.0	0.0	–2.2	0.0252018	AT2G02060.1, homeodomain-like superfamily protein
1253	1239.3	234.3	–2.2	2.07E-20	AT3G15510.1, NAC domain protein 2 (ATNAC2, ANAC056, NARS1, NAC2)
12763	2287.3	389.3	–2.4	1.1E–26	AT4G35900.1, basic-leucine zipper (bZIP) transcription factor (FD, FD-1, atbzip14)
24705	62.0	6.7	–2.4	0.001584093	AT4G08150.1, KNOTTED-like from *Arabidopsis thaliana* (KNAT1, BP, BP1)
28684	13.3	0.3	–2.4	0.011651539	AT2G45650.1, AGAMOUS-like 6 (AGL6)
18385	638.3	99.7	–2.5	2.28E-26	AT5G13180.1, NAC domain protein 83 (ANAC083, VNI2, NAC083)
9629	258.7	36.0	–2.5	0.00000013	AT5G65210.1, bZIP transcription factor family protein (TGA1)
18569	186.7	25.7	–2.5	6.88E-08	AT4G18960.1, MADS-box transcription factor family protein (AG)
26735	18.7	0.0	–2.7	0.005364437	AT3G20640.1, bHLH DNA-binding protein
14725	96.3	9.3	–2.9	0.00000184	AT5G65640.1, bHLH protein 93 (bHLH093)
35345	15.3	0.0	–3.0	0.002240693	AT3G30530.1, basic leucine-zipper 42 (ATBZIP42, bZIP42)
8735	20.7	0.3	–3.1	0.001136243	AT1G29160.1, Dof-type zinc finger DNA-binding family protein
27860	204.3	17.3	–3.2	5.17E-14	AT3G04030.3, homeodomain-like superfamily protein
28117	278.7	23.7	–3.3	1.17E-19	AT3G62420.1, basic region/leucine zipper 53 (ATBZIP53, BZIP53)
27104	2479.0	192.0	–3.5	5.12E-105	AT5G14010.1, C2HC zinc finger protein (KNU)
35817	23.7	0.0	–3.8	0.0000713	AT4G39250.1, RAD-like 1 (ATRL1, RSM2, RL1)

*RNA samples were compared using RNA-seq. Thirty-eight candidate transcription factor genes had higher transcript abundance in the skin sample (>2 log_*2*_fold change), while 18 had higher transcript abundance in the flesh sample (>-2 log_*2*_fold change). Gene model number is abbreviated with full number shown in **Supplementary Table S2**. Data shown are genes with differential expression with a cut-off value of greater than 2 log_*2*_fold.*

A number of bHLH TF gene models were strongly differential in the two tissues, including *phytochrome-interacting factor 7* and *phytochrome-interacting factor 3-like 5* (*PIL5*) which were in greater abundance in skin. Two bHLH gene models were more highly represented in flesh. Other TF classes represented included NACs, WRKYs, ERFs, and bZIPs, all classes with examples of potential association with anthocyanin regulation. Also of note was the homeodomain *Anthocyaninless2* (*ANL2*)-like gene model (36826), with a 3.9 log_2_fold differential. This gene controls anthocyanin accumulation in sub-epidermal tissues in Arabidopsis (Kubo et al., 1999) and may act upstream of the MBW complex (Kubo et al., 2008), although this remains unresolved.

### *MYBA* Transcript Abundance Correlates With Anthocyanin Production

Blueberries (highbush and rabbiteye) produce anthocyanins in a range of tissues. In fruit, anthocyanins are produced during ripening and are limited to the skin, while the flesh is essentially acyanic (**Figure 5A**). Mature rabbiteye blueberry leaves are uniformly green in color; however, newly formed leaves sometimes have a pinkish red blush around the leaf margins (**Figure 5B**). Blueberry flowers commonly have white or weakly blushed petals (**Figure 5C**). Analysis of anthocyanin content by HPLC identified a variety of anthocyanins in fruit skin, which were predominantly purple-blue trihydroxylated delphinidin-derived (**Figure 5D**). In contrast, the anthocyanin profile of red leaves was much simpler, consisting of cyanidin 3-*O*-galactoside and cyanidin 3-*O*-glucoside, which were not detected in green leaves (**Figure 5E**).

The RNA-seq data showed that *MYBA* expression was highly differential between the pigmented skin and acyanic flesh of the berry. To more widely examine the association of *MYBA* expression with anthocyanin accumulation patterns in blueberry, we conducted qPCR analysis and included the key biosynthetic gene *DFR* (**Figures 5F,G**). The anthocyanin-rich berry skin had the highest relative expression of *DFR* and *MYBA*, and anthocyanin-containing red leaves also displayed higher *DFR* and *MYBA* expression than green leaves. Expression levels of the genes in red leaves were over 6 (*DFR*) and 150 fold (*MYBA*) lower than in the skin, possibly reflecting the considerably lower concentrations of total anthocyanins. Whilst the relative expression of *MYBA* was significantly higher in red leaves than in green leaves, its relative amount was similar to that in the acyanic berry flesh. This might indicate that *MYBA* does not regulate anthocyanin accumulation in the leaves, or might suggest that this relatively low level of *MYBA* expression is not sufficient to drive the pathway flux toward anthocyanin accumulation in blueberry flesh.

## Discussion

In dicot species characterized to date, R2R3MYBs of SG6 are the key activators of the flavonoid biosynthetic pathway for the production of anthocyanin pigments in fruit. Nevertheless, previous studies on regulation of the anthocyanin pathway in the *Myrtilis* section of *Vaccinium* have suggested that R2R3MYBs of a clade of PA regulators (separate to either SG5 or SG6) fulfill this role in these species. However, our collective findings presented here on blueberry taxa (*Vaccinium* section *Cyanococcus*) agree with the data for other dicots, and identify the SG6 R2R3MYB MYBA as an anthocyanin regulator and the probable central activator of berry skin pigmentation.

In phylogenetic analysis, MYBA separated clearly in the SG6 clade, while the previously identified *Vaccinium* sequences were placed in other clades. Moreover, the deduced amino acid sequence of *MYBA* had the conserved motifs identified for SG6 proteins. The *MYBA* cDNA sequence was able to functionally substitute for the endogenous R2R3MYB anthocyanin regulator of the model species *A. majus*. Additionally, MYBA expression correlated with anthocyanin production and MYBA activated promoters of the anthocyanin biosynthetic gene *DFR*, both from blueberry and from other species (Arabidopsis and apple). This activation was (variably) increased when MYBA was co-introduced with a heterologous bHLH partner, and MYBA together with a heterologous bHLH partner gave effective induction of anthocyanin production in *N. benthamiana* leaves. These data also provide strong evidence for MYBA acting within a MYB-bHLH-WDR complex as shown for similar anthocyanin regulators of other species.

Confirming that MYBA is the central, coordinating activator of blueberry fruit skin pigmentation will require additional genetic evidence. However, we did not find evidence supporting a role for other types of R2R3MYB in anthocyanin pathway activation. There were no SG5 candidate sequences with significant differential expression in the skin. The other R2R3MYB sequences that did have higher abundance in the skin transcriptome are candidate sequences for flavonoid pathway repressors, which in other species are known to interact with the MBW complex to modulate anthocyanin production. Other transcripts corresponding to TFs with a characterized role in negatively regulating flavonoid biosynthesis were also more abundant in the skin, such as for *squamosa promoter binding protein-like* sequences.

We do not know why SG6 R2R3MYB anthocyanin regulators have not been identified previously in *Vaccinium* species. One possibility is that there is variation in the presence of SG6 genes across the genus. However, *MYBA* is present in the draft cranberry genome (Polashock et al., 2014) and is expressed (Sun et al., 2015; **Supplementary Figure S1**). As cranberry is in a different lineage to blueberry, being part of the *Oxycoccus* section of *Vaccinium*, this suggests widespread occurrence of *MYBA*-like genes in the genus. However, this does not preclude the *Myrtillis* lineage from having divergent regulatory factors. Alternatively, the SG6 genes could have narrow windows of active expression during berry development, so that previous transcriptome studies have not been at the appropriate developmental stage.

All dicot species studied in detail to date have a small multi-gene family for the SG6 anthocyanin regulatory genes, with different family members having distinct expression profiles to generate tissue-specific or developmental variations in pigmentation (Schwinn et al., 2006; Walker et al., 2007; Albert et al., 2011, 2015). In grape, berry color is primarily controlled by *VvMYBA1* and *VvMYBA2*, which exist within a complex locus on chr2 that contains 4 SG6 *R2R3MYB* genes (Walker et al., 2007), while vegetative pigmentation patterns are determined by a locus on chr14 that contains three SG6 MYB genes, with *VvMYBA7* linked to bud coloration and *VvMYBA6* to pigmented leaves and tendrils (Matus et al., 2017). Our PCR and transcriptome analyses for blueberry identified only a single transcript type encoding a functional SG6 protein. However, as *MYBA* was relatively lowly expressed in blueberry leaf tissues (**Figure 5E**), it is expected that another MYB gene family member is responsible for controlling pigmentation in vegetative tissues. In petunia for example, a specific gene family member *PURPLE HAZE* is light-regulated in vegetative tissues and in immature flower buds, conferring pigmentation to stems and leaves and a blush phenotype to flowers (Albert et al., 2011), with additional MYB genes responsible for controlling full flower color during development (Quattrocchio et al., 1999). In blueberry, young or stressed leaves produce anthocyanins (**Figure 5B**) and immature berries are also blushed with anthocyanin on the sun-exposed side, and only later develop fully pigmented fruit during maturation (Zifkin et al., 2012). Interestingly, the anthocyanins detected in blueberry leaves were cyanidin-based (**Figure 5E**), while berry skin has predominantly trihydroxylated anthocyanins (**Figure 5D**). In grape, the vegetative MYB genes were not effective at regulating *F3′5′H*, resulting in cyanidin-based pigments, while the berry *VvMYBA1* gene strongly activated this gene, resulting in trihydroxylated anthocyanins (Matus et al., 2017). Thus, it seems likely that a small family of R2R3-MYB genes controls anthocyanin biosynthesis in blueberry.

Whether the skin, flesh, or both skin and flesh are anthocyanin pigmented, and the strength of any such pigmentation, is a trait that varies across the *Vaccinium* genus, and that is of interest to plant breeding programs targeting novel commercial characters. In this regard, MYBA is a good candidate sequence for marker development for accelerated breeding approaches. The RNA-seq data will provide an additional resource for a comparison of gene expression between skin and flesh in blueberry. The other TFs identified with strongly enhanced expression in the skin could also be useful candidates for further examination for an association with differing pigmentation intensities.

## Author Contributions

KS, BP, RE, KD, AA, and NA contributed to project planning and conducted sequence analysis. KS cloned MYBA. JT provided germplasm and expertise in *Vaccinium* sp. KS and EG conducted biolistic transformations. BP performed the *Nicotiana* transformations and promoter activation assays. SC and AD conducted metabolite assays and analysis. BW carried out bioinformatics. KS, BP, KD, RE, and NA wrote the manuscript. All authors contributed to manuscript editing.

## Conflict of Interest Statement

The authors declare that the research was conducted in the absence of any commercial or financial relationships that could be construed as a potential conflict of interest.

## Footnotes

1 http://www.igbquickload.org/blueberry/V_corymbosum_Aug_2015/

2 https://www.vaccinium.org/analysis/36

## Supplementary Material

FIGURE S1 *MYBA* orthologs from rabbiteye blueberry and cranberry. Nucleotide alignment of the coding sequence of *MYBA* from highbush blueberry (*Vaccinium corymbosum; Vc*) and orthologs from rabbiteye blueberry (*V. ashei; Va*) and cranberry (*V. macrocarpon; Vm*). Sequence identity between *VcMYBA* and the orthologs from rabbiteye blueberry and cranberry are 97 and 95%, respectively. The putative start codon that has a strong Kozak sequence is indicated; the first ATG has a weak Kozak sequence and is not in-frame in rabbiteye blueberry.

FIGURE S2 Alignment of the deduced amino acid sequence of VcMYBA with those of selected flavonoid-related R2R3MYBs. The deduced amino acid sequence of VcMYBA was aligned with representative R2R3MYB genes from SGs 4, 5, 6 and the PA1-type clade using MUSCLE. Sequences from *Vaccinium* species are in bold. The C-terminal motifs that define each SG (Stracke et al., 2001) are indicated, as are the bHLH interaction motif (Zimmermann et al., 2004), the “ANDV” motif present within the MYB domain of SG6 genes, and the TLLLFR repression domain present in some SG4 MYB repressors. At, *Arabidopsis thaliana*; Vc, *Vaccinium corymbosum*; Vv, *Vitis vinifera*; Vu, *Vaccinium ulignosum.*

FIGURE S3 MYBA induces anthocyanin biosynthesis in blueberry petals. Petals from *V. corymbosum* were biolistically transformed with either *35S:GFP-ER* alone or *35S:VcMYBA + 35S:GFP-ER*. Transformed cells were identified by GFP fluorescence, observed with blue light. Transformation with *VcMYBA* induced pink pigmentation in transformed cells, while *GFP-ER* alone did not.

TABLE S1 Sequences of DNA primers used.

TABLE S2 Full list of differentially expressed genes in the skin and flesh of mature-stage blueberry fruit, with a differential greater than 2 log_2_fold.
