# Supplementary material for: MYBA From Blueberry (Vaccinium Section Cyanococcus) Is a Subgroup 6 Type R2R3MYB Transcription Factor That Activates Anthocyanin Production
Source: Front Plant Sci. 2018 Sep 11;9:1300. doi: 10.3389/fpls.2018.01300 (PMC6141686; doi:10.3389/fpls.2018.01300)
Supplement: FIGURE S1 — MYBA orthologs from rabbiteye blueberry and cranberry. Nucleotide alignment of the coding sequence of MYBA from highbush blueberry (Vaccinium corymbosum; Vc) and orthologs from rabbiteye blueberry (V. ashei; Va) and cranberry (V. macrocarpon; Vm). Sequence identity between VcMYBA and the orthologs from rabbiteye blueberry and cranberry are 97 and 95%, respectively. The putative start codon that has a strong Kozak sequence is indicated; the first ATG has a weak Kozak sequence and is not in-frame in rabbiteye blueberry. [file Data_Sheet_1.PDF]

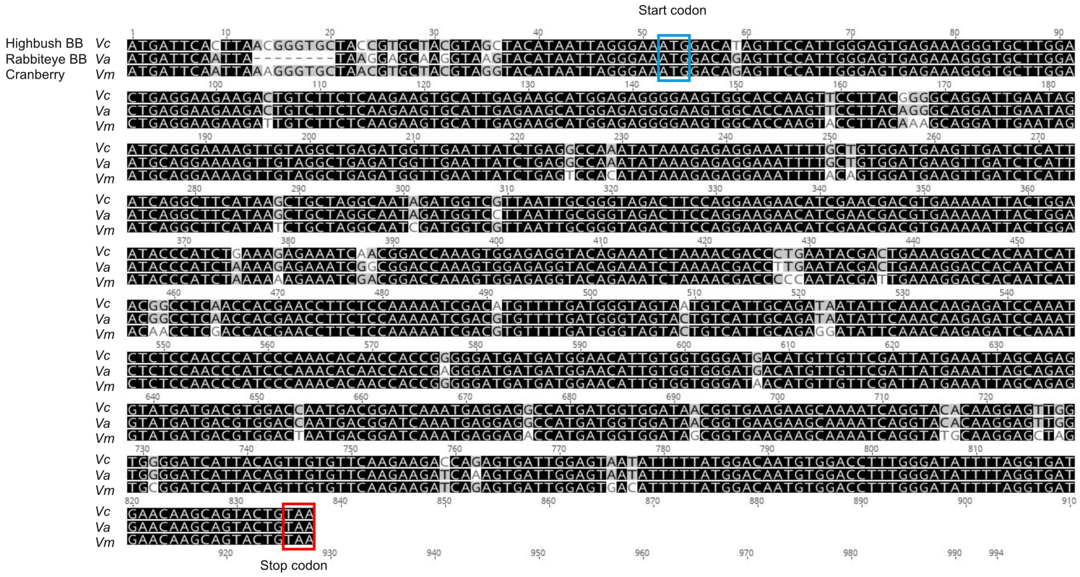

Figure S1. ***MYBA*** **orthologues from rabbiteye blueberry and cranberry**. Nucleotide alignment of the coding sequence of *MYBA* from highbush blueberry (*Vaccinium corymbosum; Vc*) and orthologues from rabbiteye blueberry (*V. ashei; Va*) and cranberry (*V. macrocarpon; Vm*). Sequence identity between *VcMYBA* and the orthologues from rabbiteye blueberry and cranberry are 97% and 95%, respectively. The putative start codon that has a strong Kozak sequence is indicated; the first ATG has a weak Kozak sequence and is not in-frame in rabbiteye blueberry.

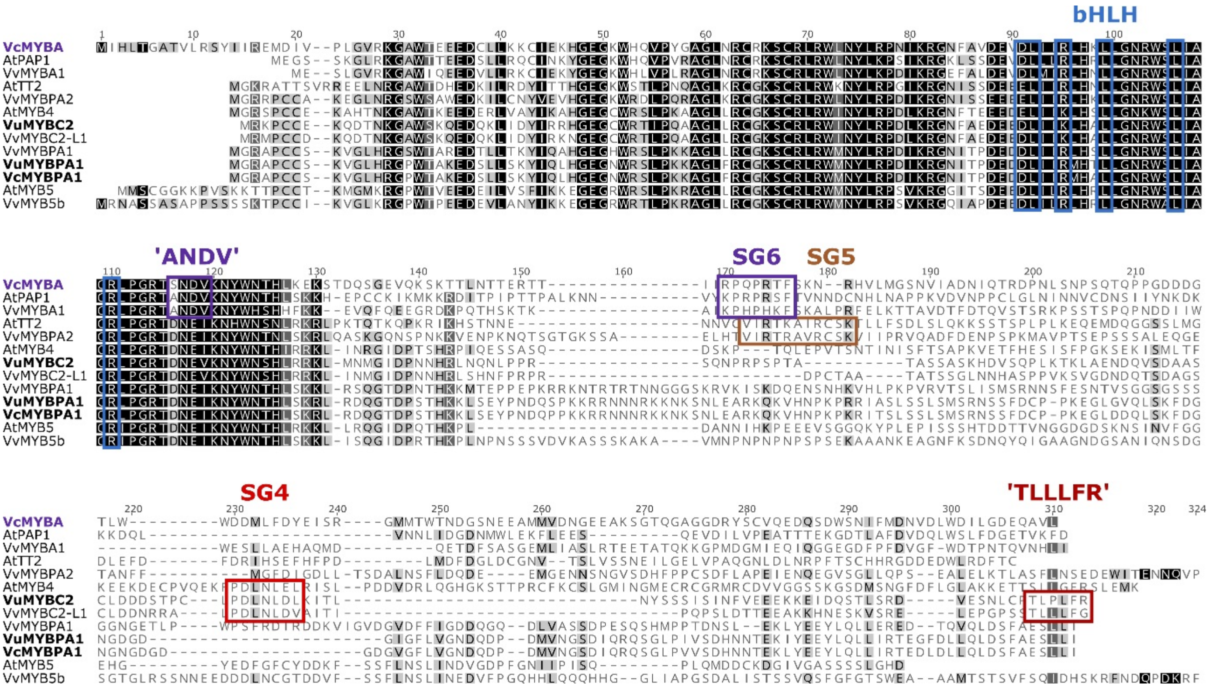

Figure S2 Alignment of the deduced amino acid sequence of VcMYBA with those of selected flavonoid-related R2R3MYBs. The deduced amino acid sequence of VcMYBA was aligned with representative R2R3MYB genes from subgroups 4, 5, 6 and the PA1-type clade using MUSCLE. Sequences from *Vaccinium* species are in bold. The C-terminal motifs that define each subgroup (Stracke et al 2001) are indicated, as are the bHLH interaction motif (Zimmerman et al 2004), the 'ANDV' motif present within the MYB domain of SG6 genes, and the TLLLFR repression domain present in some SG4 MYB repressors. Abbreviations: At *Arabidopsis thaliana*, Vc *Vaccinium corymbosum*, Vv *Vitis vinifera*, Vu *Vaccinium ulignosum.*

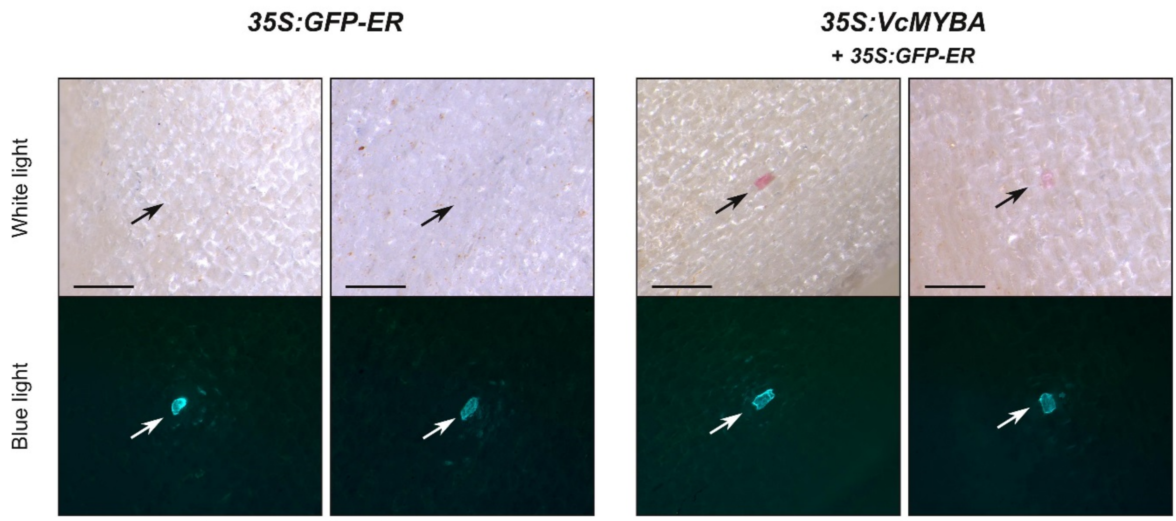

Figure S3 MYBA induces anthocyanin biosynthesis in blueberry petals

Petals from *V. corymbosum* were biolistically transformed with either *35S:GFP-ER* alone or *35S:VcMYBA + 35S:GFP-ER*. Transformed cells were identified by GFP fluorescence, observed with blue light. Transformation with *VcMYBA* induced pink pigmentation in transformed cells, while *GFP-ER* alone did not.
