# Supplementary material for: MYBA From Blueberry (Vaccinium Section Cyanococcus) Is a Subgroup 6 Type R2R3MYB Transcription Factor That Activates Anthocyanin Production
Source: Front Plant Sci. 2018 Sep 11;9:1300. doi: 10.3389/fpls.2018.01300 (PMC6141686; doi:10.3389/fpls.2018.01300)
Supplement: TABLE S1 — Sequences of DNA primers used. [file Table_1.DOCX]

**Supplementary Table 1**. Sequences of DNA primers used.

| Primer name | Primer sequence (5' to 3') |
| --- | --- |
| K115 | CTYATYITICGSCTYCAYARGCTBCT |
| K119 | TGGTCIYTRATTGCWGGWAGA |
| SP | GACTCGAGTCGACATCGA |
| K342 | AGCGTAACAATCGATGGA |
| K345 | GGAGAAGGTTCGTGGTTGAG |
| K346 | CCATCATGGCCTCCTCAT |
| K351 | AGTCGAATTCATGGACATAGTTCCATTG |
| K352 | ACGTTCTAGAAGCGTAACAATCGATGGA |
| VcMYBA - F | CTCTCCAACCCATCCCAAAC |
| VcMYBA - R | ACCGTTATCCACCATCATGG |
| VcDFR - F | CACTGAGTTTAAGGGGATTCCTAAGG |
| VcDFR - R | CCCTTCTCCCTACAAGTGTCAATGG |
| VcActin - F | AGGCTAACCGTGAGAAGATGAC |
| Vc Actin - R | AGAGTCCAGCACGATTCCAG |
